# Supplementary material for: NtMYB3, an R2R3-MYB from Narcissus, Regulates Flavonoid Biosynthesis
Source: Int J Mol Sci. 2019 Nov 1;20(21):5456. doi: 10.3390/ijms20215456 (PMC6862390; doi:10.3390/ijms20215456)
Supplement: Supplementary file 1 [file ijms-20-05456-s001.pdf]

NtMYB3, an R2R3-MYB from Narcissus, Regulates Flavonoid Biosynthesis

Muhammad Anwar [1,†], Weijun Yu [1,†], Hong Yao [1], Ping Zhou [1], Andrew C. Allan [2,3], and Lihui Zeng [1,*]

[1] College of Horticulture, Fujian Agriculture and Forestry University, Fuzhou 35002, China; anwar_uaar@yahoo.com (M.A.); ywj973618858@hotmail.com (W.Y.); yaohong116@hotmail.com (H.Y.); zhoup1249897684@hotmail.com (P.Z.)

2 The New Zealand Institute for Plant & Food Research, Mt Albert Research Centre, Private Bag 92169, Auckland, 1025 New Zealand; andrew.allan@plantandfood.co.nz

[3] School of Biological Sciences, University of Auckland, Private Bag 92019, Auckland 1142, New Zealand

* Correspondence: lhzeng@hotmail.com Tel.: +86-591-83789450

† These authors contributed equally to this work.

Supplementary Material

Supplemental Table 1. Analysis of putative cis-acting elements in*NtFLS*promoter.

Motif	Function	Strand	Position[a]	Position(5′ - 3′)
5′ UTR Py-rich stretch stretch	*cis*-acting element conferring high transcription levels	+	−552	TTTCTCTC TCTCTC
TATA-box	core promoter element around -30 of transcription start	+	−831、−884、−895、−908、900	TATA
			−883、−909	ATATAT
			−908	TATATA
		−	−63	TATA
			−63	TATAA
			−908	TATATAA
CAAT-box	common cis-acting element in promoter and enhancer regions	+	−446	CCAAT
			−627、−832、−466	CAAT
			−515	CAAAT
		−	−910、−860、	CAAT
			−619、−650、−331	CAAAT
3-AF1 binding site	Light responsive element	−	−421	TAAGAGA GGAA
Box II	part of a light responsive element	+	−213	CCACGTGG C
G-Box	cis-acting regulatory element involved in light responsiveness	−	−215	CACGTG
G-box	cis-acting regulatory element involved in light responsiveness	+	−81	CACGTG
TCT-motif	part of a light responsive element	+	−402	TCTTAC
ACE	cis-acting element involved in light responsiveness	−	−107	ACTACGTT GG
Box I	Light responsive element	+	−199	TTTCAAA
Myb-binding site	MYB combination element	+	−682	CAACAG
MYB	MYB combination element	+	−682	CAACAG

ABRE	cis-acting elemen involved in the abscisic acid responsiveness	+	−215	ACGTG
		−	−106、−82	ACGTG
ABRE2		−	−214	CCACGTGG
ABRE3a		−	105	TACGTG
ABRE4		+	105	CACGTA
		−	−140	CAACCA
P-box	Gibberellin response element	−	−354	GACCAAA CTCGT
O2-site	cis-acting regulatory element involved in zen metabolism regulation	−	−532	GATGATAT GC
TC-rich repeats	cis-acting element involved in defense and stress responsiveness	+	−497	ATTCTCTA AC
		+	−407	GTTTTCTT AC
CAT-box	cis-acting regulatory element related to meristem expression	+	−313	GCCACT
Skn-1_motif	*cis*-acting regulatory element required for endosperm expression	−	−825	GTCAT

Supplemental Table 2. List of primers used in this studies.

**Name**	**Forward primer (5' to 3')**	**Reverse primer (5' to 3')**	**Note**
MYB3	AATATGGGTAGGTCTCCTTGTT GTG	GATTACAGGACACGCAAAGTAAATCTA	Cloning of full length of ORF
pSAK-*MYB3*	TGGATCCAAAGAATTCAATAT GGGTAGGTCTCCTTGTTGTG	TACTCTCGAGAAGCTTGATTACAGGAC ACGCAAAGTAAATCTA	Vector construction
QRT *NtCHS*		AAATTGCCTCTCTTGAGATCGG ACCTATAATGACCGCGGCTG	
NtCHI	TGGGTAGGTCTCCTTGTT	CAACGTTGACAACATCAGGC	
NtF3H	ACTCCGGATGGCTAAGGACTG TG	CCTTGGTTAAGGCCTCCTTC	
NtFLS	GAAATCCTCCGATCCAGTGA ACAGGGTGAAGTGGTCCAAG	TCCCTGTAGGAGGGAGGATT	
NtLAR	GAACTTGAAGGGAAAAGGGG TCAAGGTCCTTTACGCCATC	ACGAACCTGCTTCTCTTTGG	qRT-PCR analysis
NtANR	CATTTGACTTTCCCAAACGC GGGAATGAAGCTCACTACAGC	ATTGGGCTTTTGAGTTGTGC	
NtDFR	GAGTGCATTGGATGCCTTTT TGGCGTTGAAGCTCATACTG	ACTCCGGCCATTTCTCTTGG	
NtUFGT	AATGGAACTGGAATGGTCAAG GC	CCAGCTCCATTAGGTCCTTG	
NtANS		GGAATTAGGCACACACTTTGC	
NtACT		TGCCAGATCTTCTCCATGTCATCCCA	
